# Alterations in the functional connectivity of thalamic subregions after basal ganglia stroke

**DOI:** 10.3389/fneur.2025.1584290

**Published:** 2025-06-06

**Authors:** Qiansheng Cheng, Shoufeng Liu, Junling Wang, Yajing Wang, Bihui Han, Lichen Wang, Song Jin

**Affiliations:** ^1^Department of Radiology, Tianjin Huanhu Hospital, Tianjin, China; ^2^Huanhu Hospital Affiliated to Tianjin Medical University, Tianjin, China; ^3^Department of Neurology, Tianjin Huanhu Hospital, Tianjin, China

**Keywords:** basal ganglia stroke, brain, functional connectivity, functional magnetic resonance imaging, thalamic subregions

## Abstract

**Background:**

Motor and cognitive impairments are common symptoms of basal ganglia (BG) stroke, although the underlying neurobiological mechanisms remain unclear. Therefore, we aimed to explore the alterations in functional connectivity (FC) between thalamic subregions post-BG stroke via resting-state functional magnetic resonance imaging (rs-fMRI) measurements.

**Methods:**

This cross-sectional study compared 40 patients with BG stroke and 35 healthy controls (HCs). Seed-based FC analysis was performed for 14 thalamic subregions. Correlations between FC changes and Fugl–Meyer Assessment (FMA)/Mini-Mental State Examination (MMSE) scores were assessed.

**Results:**

Patients exhibited hyperconnectivity between the left thalamic subregion connected with the sensory cortex (SC_thalamus) and left precuneus (*t* = 3.97, *p*FWE = 0.041) and the right SC_thalamus–left angular gyrus (*t* = 4.50, *p*FWE = 0.032). Hypoconnectivity emerged between the left thalamic subregion connected with the prefrontal cortex (PFC_thalamus) and right supramarginal gyrus (*t* = −5.54, *p*FWE = 0.015), left thalamic subregion connected with the temporal cortex (TC_thalamus) and right postcentral gyrus (*t* = −4.95, *p*FWE = 0.022), and right thalamic subregion connected with the primary motor cortex (M1_thalamus) and right medial suprafrontal gyrus (*t* = −5.62, *p*FWE = 0.012). FC strength between the right M1_thalamus and right medial suprafrontal gyrus was positively correlated with FMA (*r* = 0.484, *p*FDR = 0.033), while left PFC_thalamus–right supramarginal connectivity predicted MMSE performance (*r* = 0.490, *p*FDR = 0.021).

**Conclusion:**

BG stroke disrupts thalamocortical circuitry at subregional levels, with distinct FC patterns linking to motor/cognitive deficits. These network-level insights may guide targeted neuromodulation therapies. The identified FC alterations could serve as biomarkers for monitoring recovery and personalizing interventions to improve post-stroke rehabilitation outcomes.

## 1 Introduction

Stroke-associated disability and mortality rates are extremely high among middle-aged and older adults, posing a huge burden on public health (1). Blood supply to the basal ganglia (BG) is abundant, where a cerebral infarction usually occurs. The BG plays a crucial role in coordinating complex movements and regulating advanced cognitive functions (2, 3). Patients with BG stroke frequently exhibit persistent motor and cognitive deficits (4), reflecting large-scale network dysfunction rather than isolated focal impairments (5). Notably, these deficits arise not only from local ischemic damage but also through diaschisis—functional disruptions in remote yet interconnected brain regions (6–8). In addition, the abnormal function of cortical–BG–thalamocortical circuits is believed to cause cognitive decline in patients with BG stroke (9). However, conventional neuroimaging paradigms often treat the thalamus as a homogeneous entity, averaging blood oxygen level-dependent (BOLD) signals across its subnuclei. This approach obscures critical FC patterns within specific thalamocortical circuits and limits the identification of subregion-specific diaschisis mechanisms (10). Given that distinct thalamic subregions project to functionally specialized cortical networks (e.g., motor vs. prefrontal regions) (11), a subregional analysis is essential for uncovering circuit-level disruptions underlying post-stroke deficits.

Anatomically, the thalamus can be parcellated into seven distinct subregions based on cortico-thalamic connectivity profiles derived from the probability fiber tracing algorithm (11). Building upon this framework, we defined 14 bilateral thalamic subregions as regions of interest (ROIs). Related studies have shown that secondary damage manifests in the thalamus far from the infarcted site after cerebral infarction (12, 13), with ipsilateral thalamic volume loss correlating with cognitive outcomes (14). Preliminary functional investigations have also reported increased thalamo-cerebellar connectivity in stroke patients compared to healthy controls (HCs) (15). Nevertheless, the abovementioned studies have treated the thalamus as a whole structure, potentially masking compensatory or pathological changes specific to individual subregions. For instance, connectivity alterations in motor-related versus cognitive-related thalamic subregions may differentially predict FMA or MMSE scores—distinctions that remain unexplored.

This study investigates subregional thalamic FC alterations in patients with BG stroke using rs-fMRI. We systematically compared whole-brain, voxel-wise FC profiles of 14 thalamic subregions between patients with BG stroke and HCs and assessed correlations between aberrant FC patterns and clinical outcomes using the MMSE for cognition and the FMA for motor recovery. By moving beyond whole-thalamus analyses, our subregion-specific approach reveals (1) which thalamocortical circuits are selectively disrupted or preserved post-stroke and (2) how these disruptions map to motor versus cognitive deficits—a critical advance over prior homogeneous thalamus models. These findings offer novel insights into the neural mechanisms underlying post-stroke motor and cognitive rehabilitation.

## 2 Materials and methods

### 2.1 Participants

This prospective observational study enrolled 40 patients with acute BG stroke from the Neurology Department of Tianjin Huanhu Hospital between May 2022 and October 2023. Concurrently, 35 age-, sex-, education-, and body mass index (BMI)-matched HCs were recruited through community advertisements during the same period. The inclusion criteria were as follows: (1) unilateral, single-lesion BG stroke for the first time diagnosed by magnetic resonance imaging (MRI) within 7 days of neurological symptoms, (2) middle-aged and older adults aged 45–75 years, and (3) right-handedness interpreted using the Edinburgh Handedness Inventory. In addition, the exclusion criteria included (1) contraindications to MRI, (2) comorbidities or medications (e.g., antiepileptics and antipsychotics) that may influence the experimental outcome, and (3) cases of unqualified images.

### 2.2 Ethics statement

This study protocol was approved by the Ethics Committee of Tianjin Huanhu Hospital (approval number 2022-056) and was conducted in accordance with the Declaration of Helsinki. Written informed consent was obtained from all participants before the experiment.

### 2.3 Instrumental assessments

On the day of the MRI examination, cognitive and motor functions in the upper limbs were assessed using the MMSE and the FMA scores, respectively.

### 2.4 Data acquisition

The MR data of all participants were captured from a *3T* Prisma scanner (Siemens Healthcare, Erlangen, Germany) in the Imaging Department of Tianjin Huanhu Hospital. Participants were instructed to relax, close their eyes, and forget distracting thoughts during the MR data acquisition. High-resolution 3DT1, T2, fluid-attenuated inversion recovery (FLAIR), diffusion-weighted imaging (DWI), and rs-fMRI images were captured. High-resolution 3DT1-weighted anatomical images were acquired using a magnetization-prepared rapid-acquisition gradient-echo sequence with the following parameters: repetition time [TR], 1,560 ms; echo time [TE], 1.65 ms; inversion time [TI], 778 ms; flip angle, 8°; field of view [FOV], 256 × 256 mm^2^; matrix size, 256 × 256; slice thickness, 1 mm; and number of slices, 192. In addition, the rs-fMRI images were acquired using echo planar imaging (TR, 1000 ms; TE, 30 ms; flip angle, 70°; FOV, 220 × 220 mm^2^; matrix, 110 × 110; number of transverse slices, 64; voxel size, 2 × 2 × 2.2 mm^3^; and slice volume, 400). The whole-brain image was acquired with the phase-encoded direction from anterior to posterior.

### 2.5 Lesion probability map

**Figure 1:**
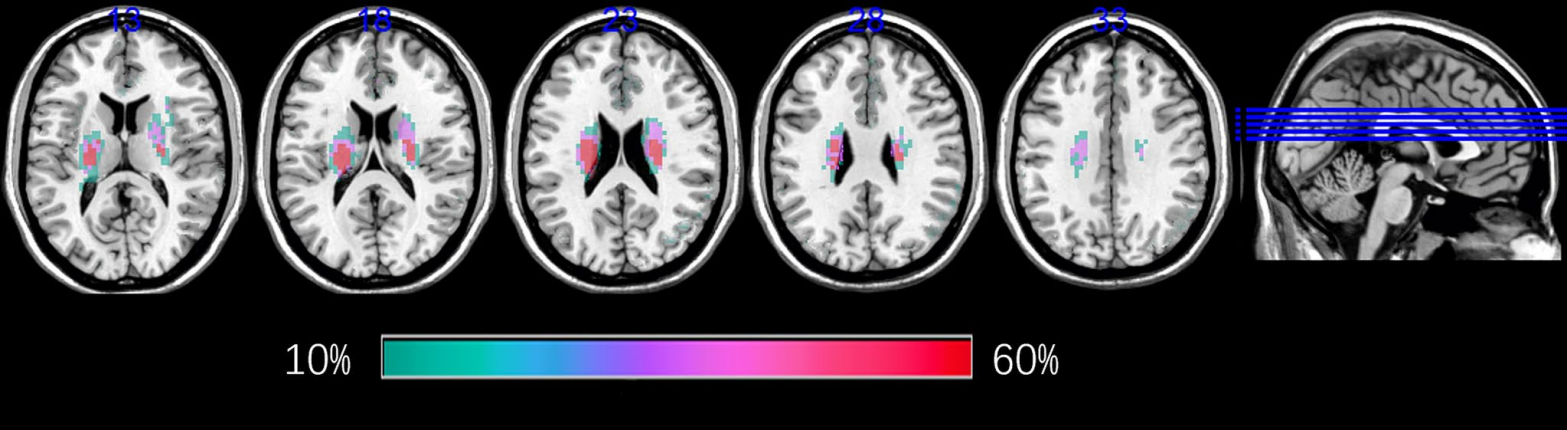
Lesion probability maps for patients with BG stroke. The heat maps corresponding to the probability of patients having a lesion in that area were overlaid on axial slices from a standard template in MNI space. BG, basal ganglia; MNI, Montreal Neurological Institute.

Lesion probability maps were constructed as follows (Figure 1): (1) manual delineation of acute BG stroke lesions on DWI using MRIcron^1^; (2) individual lesion masks were rigidly aligned with individual T1 images using six-degree-of-freedom transformations in statistical parametric mapping (SPM12, http://www.fil.ion.ucl.ac.uk/spm); (3) coregistered lesion maps were spatially smoothed with a 4-mm full-width half-maximum (FWHM) Gaussian kernel, and registration to Montreal Neurological Institute (MNI) space was performed via SPM12’s unified segmentation-normalization algorithm; (4) voxel-wise averaging of normalized binary masks (0–1 scale); and (5) thresholding at > 10% overlap (intensity > 0.1).

### 2.6 Imaging data preprocessing

**Figure 2:**
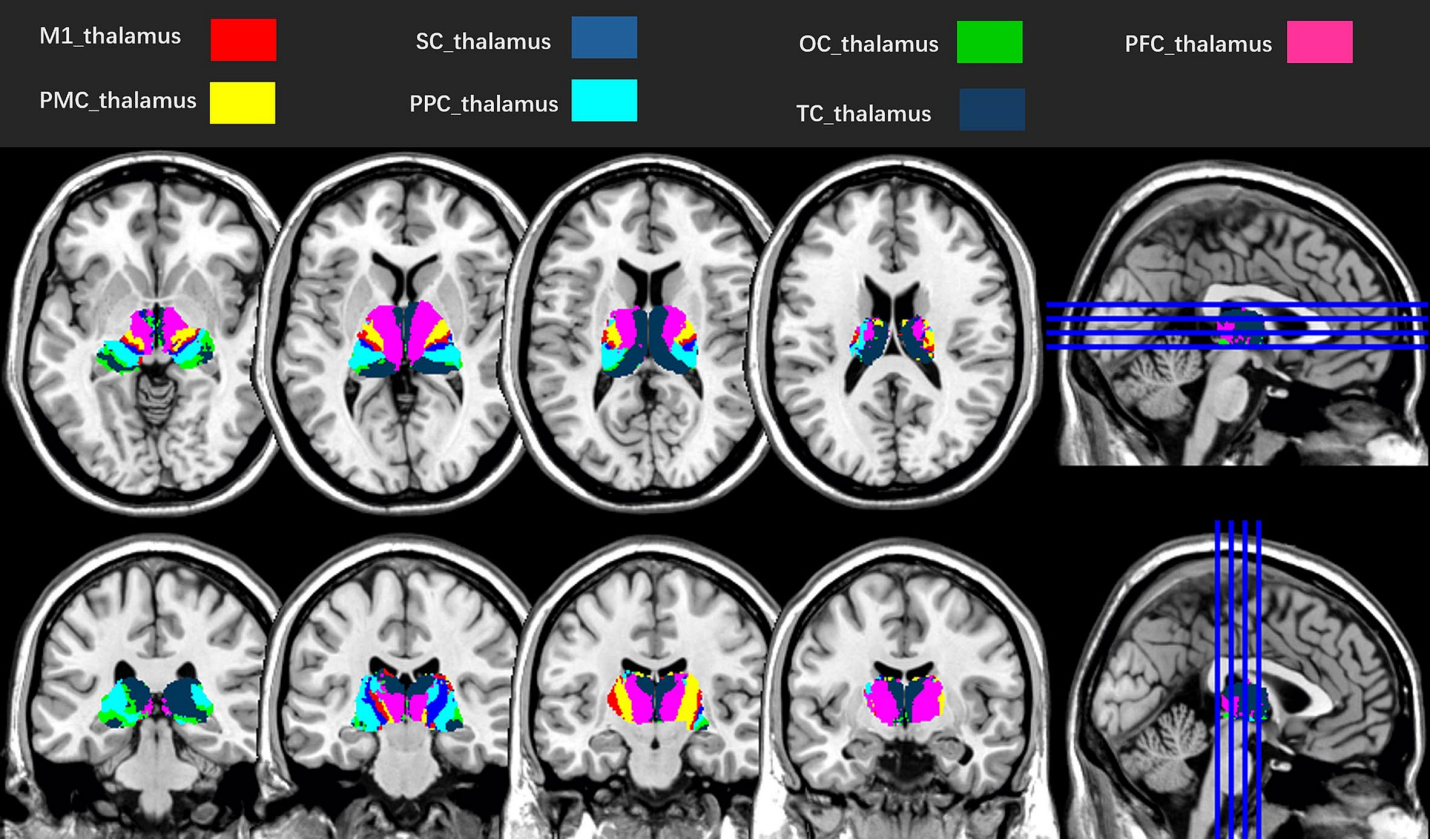
Seven subregions of the left and right thalami, according to the probability fiber tracing algorithm (11). M1_thalamus, thalamic subregion connected with the primary motor cortex; SC_thalamus, thalamic subregion connected with the sensory cortex; OC_thalamus, thalamic subregion connected with the occipital cortex; PFC_thalamus, thalamic subregion connected with the prefrontal cortex; PMC_thalamus, thalamic subregion connected with the premotor cortex; PPC_thalamus, thalamic subregion connected with the posterior parietal cortex; TC_thalamus, thalamic subregion connected with the temporal cortex.

The Data Processing Assistant for Resting-State fMRI (DPARSF, http://rfmri.org/DPARSF), based on SPM12, was adopted to preprocess the rs-fMRI images (16). In brief, the first 20 volumes were removed to allow an adaptation, retaining 380 volumes for analysis. Temporal offsets were adjusted across slices using the middle slice as a reference for slice timing correction. Images were realigned to the first volume via rigid-body transformation (six degrees of freedom). Frames with excessive head motion (translation > 2.0 mm or rotation > 2°) were removed, and participants with > 20% removed frames were excluded. The structural T1-weighted image was preprocessed using a six-parameter rigid-body transformation to correct for head motion. It was then coregistered to the mean rs-fMRI image to establish spatial correspondence. Subsequent steps included tissue segmentation into gray matter (GM), white matter (WM), and cerebrospinal fluid (CSF) using a probabilistic atlas-based approach (17), followed by non-linear registration to the MNI stereotactic space (2 × 2 × 2 mm^3^ isotropic resolution) with volumetric registration prioritized to enhance subcortical anatomical alignment (particularly for thalamic nuclei) to standardize anatomical localization across participants. Adopting the same transformed parameters, the exponentiated Lie algebra (DARTEL) algorithm was adopted for the spatial normalization of rs-fMRI images (18). Spatial smoothing was applied to the preprocessed rs-fMRI data using a Gaussian kernel with 4 mm FWHM to balance noise reduction and preservation of spatial details, aligning with common practice in rs-fMRI studies (16). The Friston 24 motion parameters (19), mean signals in the WM and CSF, and linear trends were regressed out as covariates to account for potential confounding effects. We processed the rs-fMRI images using a temporal Fourier filter ranging from 0.01 to 0.1 Hz to remove low-frequency drift and high-frequency respiration (20). Bilateral thalami were subdivided into seven functionally distinct subregions per hemisphere (M1_thalamus, SC_thalamus, thalamic subregion connected with the occipital cortex (OC_thalamus), PFC_thalamus, thalamic subregion connected with the premotor cortex (PMC_thalamus), thalamic subregion connected with the posterior parietal cortex (PPC_thalamus), and TC_thalamus) as ROIs based on the probability fiber tracing algorithm (11) (Figure 2). FC was estimated using Pearson’s correlation of the 14 bilateral ROIs and whole-brain voxels. Fisher’s r-to-z transformation normalized the FC distributions, and the resulting z-maps were analyzed.

### 2.7 Statistical analyses

IBM SPSS Statistics for Windows, version 24.0 (IBM Corp., Armonk, N.Y., United States), was used to perform all statistical analyses. Continuous data (mean ± standard deviation) and categorical data were compared using the two-sample *t*-test and chi-squared test, respectively (21). The FC differences between patients with BG stroke and HCs were tested using non-parametric permutation tests (SnPM toolbox) (22), SPM12; 5,000 permutations, two-tailed, chosen for their suitability for small-to-moderate sample sizes, with age and sex as covariates. Effect sizes for significant FC differences were calculated as Cohen’s *d*. Analyses were restricted to a GM mask using a two-threshold approach: voxel level: *p* < 0.001 (uncorrected) and cluster level: *p*FWE < 0.05 (permutation-derived cluster extent). Significant clusters surviving correction were defined as regions exhibiting altered FC in patients with BG stroke. The mean FC z-values within these clusters were extracted for subsequent clinical correlation analysis. Pearson’s correlation was used to identify the correlation of FCs with the MMSE and FMA scores in patients with BG stroke. All comparisons underwent two-tailed FDR correction (*p*FDR < 0.05), with significance determined by adjusted *p*-values.

## 3 Results

### 3.1 Demographic and clinical characteristics of participants

No significant differences were observed in demographic data between patients with BG stroke and HCs, including age (*p* = 0.248), male sex (*p* = 0.578), education level (*p* = 0.400), and BMI (*p* = 0.833) (Table 1). The FMA (16.05 ± 2.65) and MMSE (20.15 ± 2.26) scores in patients with BG stroke followed a normal distribution.

**Table 1:** Demographic and clinical characteristics of patients with basal ganglia stroke (*n* = 40) and healthy controls (*n* = 35).

Characteristics	BG stroke patients (*n* = 40)	Healthy controls (*n* = 35)	*p*-value
Age (yrs)	60.95 ± 5.57	59.43 ± 5.73	0.248^a^
Male (*n*, %)	22 (55.0)	17 (48.6)	0.578^b^
Education level (yrs)	10.07 ± 1.70	9.74 ± 1.69	0.400^a^
BMI (kg/m^2^)	25.29 ± 1.07	25.23 ± 1.13	0.833^a^
MMSE (points)^*^	20.15 ± 2.26	-	
FMA (points)^*^	16.05 ± 2.65	-	

^*^The MMSE and FMA surveys were not applicable to healthy controls. BG, basal ganglia; BMI, body mass index; MMSE, Mini-Mental State Examination; FMA, Fugl–Meyer Assessment.

a Two-sample t-test.

b Chi-squared test.

### 3.2 FC alterations

**Figure 3:**
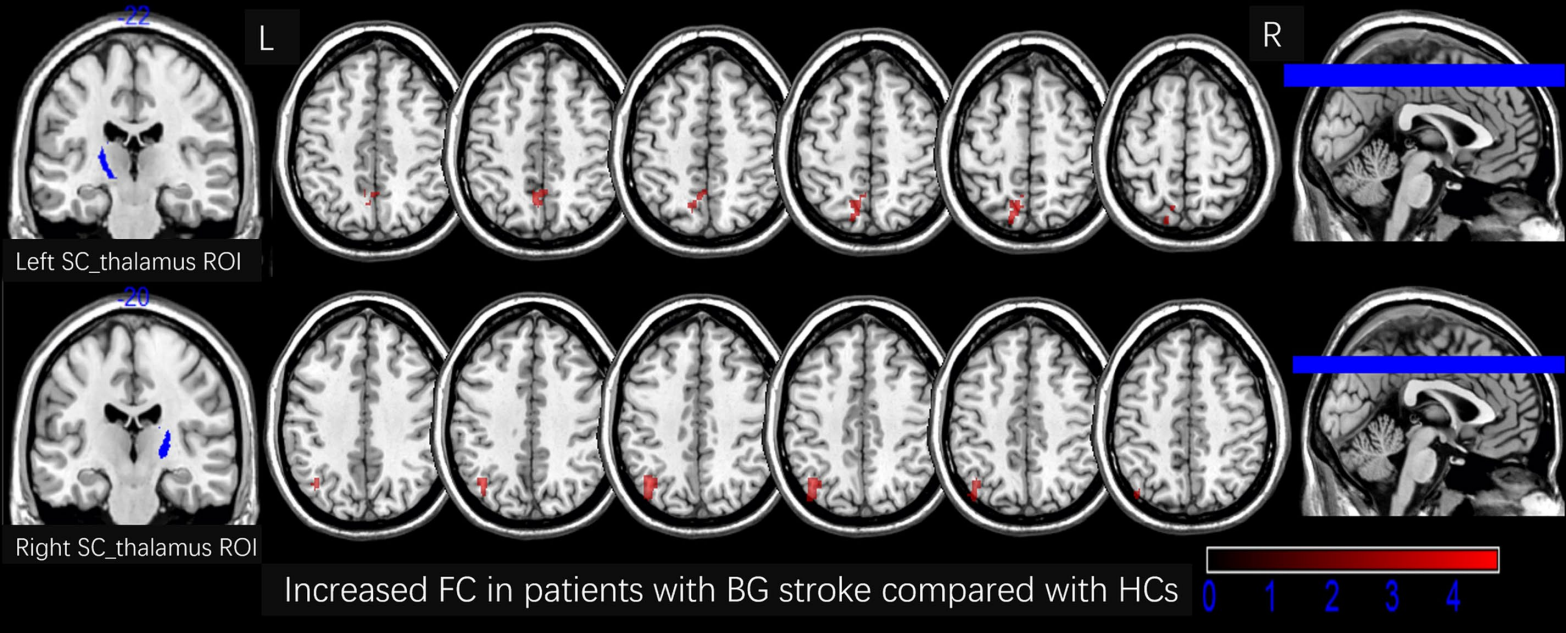
Increased FC in patients with BG stroke compared to HCs (corrected by FWE at cluster level following 5,000 permutations). The first row shows the left SC_thalamus as the ROI, with red areas in transverse sections indicating an increased FC in the left precuneus. The second row shows the right SC_thalamus as the ROI, with red areas in transverse sections indicating an increased FC in the left angular gyrus. L, left; R, right; FC, functional connection; ROI, region of interest; HCs, healthy controls; SC, sensory cortex; BG, basal ganglia; FWE, family-wise error.

**Figure 4:**
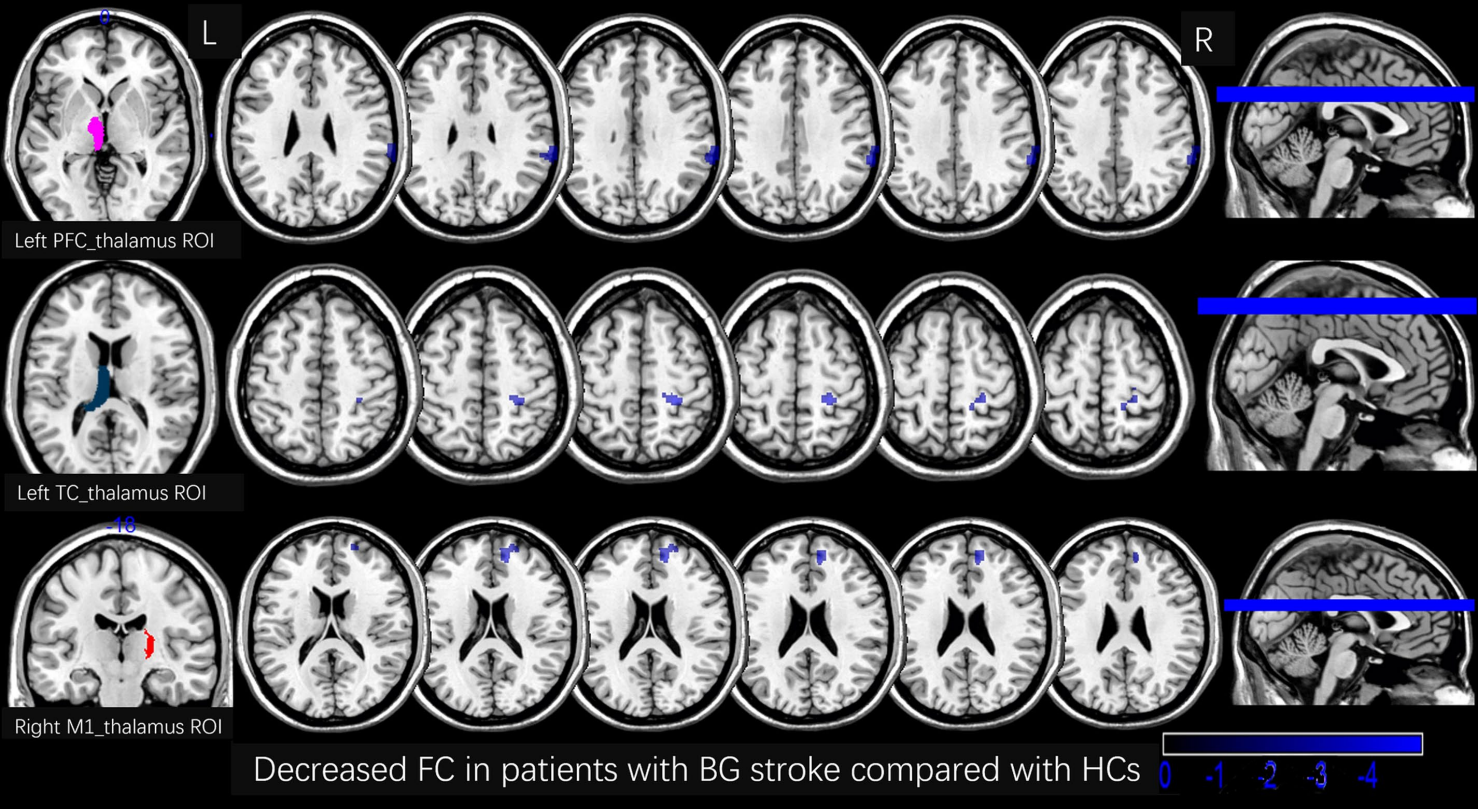
Decreased FC in patients with BG stroke compared to HCs (corrected by FWE at cluster level following 5,000 permutations). The first row shows the left PFC_thalamus as the ROI, with blue areas in transverse sections indicating decreased FC in the right supramarginal gyrus. The second row shows the left TC_thalamus as the ROI, with blue areas in transverse sections indicating decreased FC in the right postcentral gyrus. The third row shows the right M1_thalamus as the ROI, with blue areas in transverse sections indicating decreased FC in the right medial suprafrontal gyrus. L, left; R, right; FC, functional connection; ROI, region of interest; HCs, healthy controls; PFC, prefrontal cortex; BG, basal ganglia; FWE, family-wise error.

Table 2 and Figures 3, 4 show the FC alterations in the brain regions of patients with BG stroke and HCs with significant differences. Compared to HCs, patients with BG stroke had an increased FC between the left SC_thalamus and left precuneus (*t* = 3.97, *d* = 0.93, *p*FWE = 0.041) and between the right SC_thalamus and left angular gyrus (*t* = 4.50, *d* = 1.05, *p*FWE = 0.032), as illustrated in Figure 3. Conversely, as shown in Figure 4, patients with BG stroke exhibited a decreased FC between the left PFC_thalamus and right supramarginal gyrus (*t* = −5.54, *d* = −1.30, *p*FWE = 0.015), between the left TC_thalamus and right postcentral gyrus (*t* = −4.95, *d* = −1.16, *p*FWE = 0.022), and between the right M1_thalamus and right medial suprafrontal gyrus (*t* = −5.62, *d* = −1.32, *p*FWE = 0.012).

**Table 2:** Functional connectivity alterations in brain regions of patients with basal ganglia stroke.

Brain region	Cerebral hemisphere	MNI coordinates			Cluster size	*t*-value	*p*FWE
		x	y	z			
Left SC_thalamus (ROI)	Left	-	-	-	-	-	-
Precuneus	Left	−12	−63	54	72	3.97	0.041
Right SC_thalamus (ROI)	Right	-	-	-	-	-	-
Angular	Left	−39	−69	42	76	4.50	0.032
Left PFC_thalamus (ROI)	Left	-	-	-	-	-	-
Supramarginal	Right	63	−35	32	135	−5.54	0.015
Left TC_thalamus (ROI)	Left	-	-	-	-	-	-
Postcentral gyrus	Right	24	−33	57	81	−4.95	0.022
Right M1_thalamus (ROI)	Right	-	-	-	-	-	-
Medial suprafrontal gyrus	Right	12	48	21	64	−5.62	0.012

x, y, and z coordinates of primary peak locations in Montreal Neurological Institute (MNI) space. MNI, Montreal Neurological Institute; FWE, family-wise error; SC_thalamus, a thalamic subregion connected with the sensory cortex; ROI, region of interest; PFC_thalamus, a thalamic subregion connected with the prefrontal cortex; TC_thalamus, a thalamic subregion connected with the temporal cortex; M1_thalamus, a thalamic subregion connected with the primary motor cortex.

### 3.3 Correlational analysis

**Figure 5:**
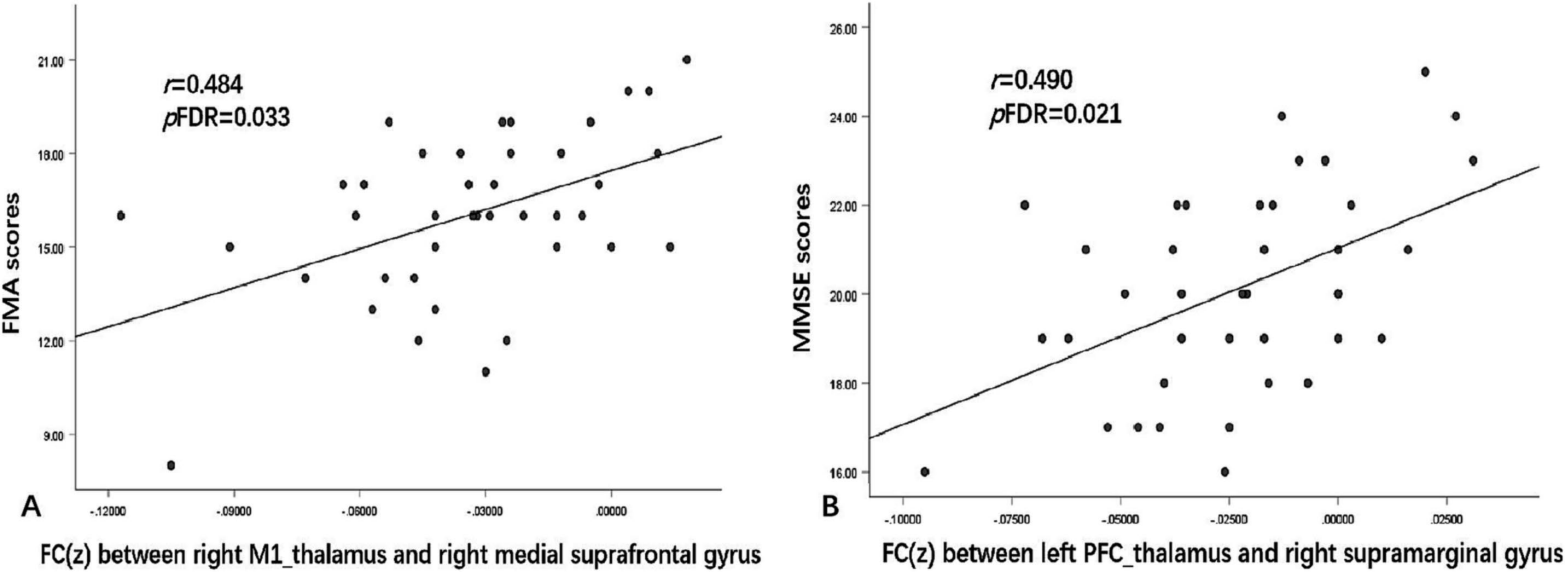
Correlations between FC and clinical symptom scores. **(A)** shows the FC values between the right M1_thalamus and right medial suprafrontal gyrus, which were positively correlated with FMA scores (*r* = 0.484, *p*FDR = 0.033). **(B)** shows the FC values between the left PFC_thalamus and right supramarginal gyrus, which were positively correlated with MMSE scores (*r* = 0.490, *p*FDR = 0.021). *r* represents the Pearson correlation; *p* represents the significance level. MMSE, Mini-Mental State Examination; FMA, Fugl–Meyer Assessment; FDR, false discovery rate.

As shown in Figure 5, a positive FC correlation was identified between the right M1_thalamus and right medial suprafrontal gyrus with the FMA scores (*r* = 0.484, *p*FDR = 0.033) and between the left PFC_thalamus and right supramarginal gyrus with the MMSE scores (*r* = 0.490, *p*FDR = 0.021). No correlations of other abnormal FC values with either FMA or MMSE scores survived FDR correction.

## 4 Discussion

Our study identified altered FC patterns between the thalamic subregions and cortical regions in patients with BG stroke, revealing significant correlations with motor and cognitive deficits. These findings suggest that post-stroke impairments may arise not only from direct ischemic damage but also through dysregulated thalamocortical network interactions. This aligns with prior evidence, demonstrating that BG stroke disrupts both sensorimotor and cognitive processing through distributed network effects (23).

The thalamus comprises functionally specialized nuclei, including the mediodorsal (MD), pulvinar, ventral lateral (VL), ventral anterior (VA), and ventral posterior (VP) nuclei. These nuclei exhibit distinct cortico-thalamic connectivity patterns: (1) The VP nucleus (which has overlapping regions with the SC_thalamus) receives somatosensory inputs and projects to the primary motor cortex, forming the SC_thalamus-M1 pathway (24–26); (2) the MD nucleus (which has overlapping regions with the PFC_thalamus), interconnected with prefrontal regions, supports higher-order cognitive functions through valuation and motivational processing (27–30); and (3) the pulvinar and MD nuclei (which have overlapping regions with the TC_thalamus) jointly mediate temporo-cortical integration for cognitive control (11, 31). Notably, VP nucleus activity precedes voluntary movements, highlighting its role in motor planning.

Patients with BG stroke exhibited an increased FC between the left SC_thalamus and left precuneus/angular gyri. These regions constitute key hubs of the default mode network (DMN), with the angular gyrus involved in semantic processing and spatial cognition (32), while the precuneus mediates self-referential processing and conscious awareness (33–35). This hyperconnectivity may reflect maladaptive compensatory mechanisms, analogous to DMN reorganization observed in post-stroke aphasia (36). Recent longitudinal studies suggest that stroke-induced DMN hyperconnectivity is often associated with persistent attentional deficits, despite offering initial compensatory benefits (37). Specifically, increased thalamo-parietal coupling may represent aberrant sensory reafference mechanisms aimed at stabilizing posture and guiding movement planning—a pattern consistent with previous reports of stroke-induced DMN hyperconnectivity (38).

Decreased FC between the left PFC_thalamus and right supramarginal gyrus is correlated with lower MMSE scores. This finding extends recent evidence, showing that prefrontal-thalamic disconnection predicts 6-month cognitive prognosis in subcortical stroke (39). As the supramarginal gyrus contributes to cognitive-emotional integration (40), this disconnection may impair executive control networks supported by prefrontal–striatal–thalamic loops (41); the MD nucleus, serving as a thalamic hub for prefrontal connectivity, likely plays a critical role in maintaining goal-directed attention and working memory.

Reduced FC between the left TC_thalamus and right postcentral gyrus aligns with prior findings of sensory processing deficits in subcortical stroke (42). The postcentral gyrus, essential for somatosensory and emotional processing (43), may lose thalamic modulation required for integrating sensory feedback with motor planning.

Diminished FC between the right M1_thalamus and medial suprafrontal gyrus is correlated with FMA scores. The positive FC-motor function correlation suggests that preserved thalamofrontal communication facilitates motor recovery, which is consistent with neuromodulation studies where TMS-enhanced M1-thalamus connectivity predicts better rehabilitation outcomes (44). This highlights thalamocortical FC as a potential biomarker for stratifying motor recovery trajectories and guiding targeted neuromodulation. The medial suprafrontal gyrus modulates motor cortex excitability (45, 46), and its decoupling from thalamic motor nuclei may disrupt cortico-subcortical circuits critical for motor recovery, as evidenced by longitudinal FC studies (47).

The structure–function relationships identified in this study provide two translational pathways: (1) Thalamocortical FC patterns could serve as predictive biomarkers, given their correlation between acute impairment (FMA) and global cognition (MMSE). This aligns with recent efforts to integrate connectomic biomarkers into stroke prognosis models (48); and (2) FC targets, such as the PFC_thalamus–supramarginal gyrus pathways, could be prioritized in non-invasive stimulation protocols, building on successful trials of thalamus-targeted transcranial direct current stimulation (tDCS) for post-stroke cognition (49).

Several limitations merit consideration in this study: (1) Volumetric registration prioritized subcortical anatomical fidelity over cortical alignment, so future studies could integrate surface-based registration (FreeSurfer) with *7 T* thalamic segmentation; (2) The rs-fMRI’s extended scan time limits bedside applicability, so abbreviated protocols should be explored; and (3) The single-center design, right-handed cohort (lacking diversity in cerebral lateralization and excluding non-right-handed individuals), and cross-sectional data require validation in larger, longitudinal cohorts with population diversity, including left-handed/ambidextrous individuals.

Future research should employ graph-theoretical and dynamic connectivity analyses (50, 51) to (1) quantify changes in global network topology (e.g., small-worldness and hub distribution); (2) track temporal fluctuations in thalamocortical coupling during recovery; and (3) integrate multi-modal data (e.g., DTI tractography and task-fMRI) to resolve structure–function decoupling.

## 5 Conclusion

This study establishes that BG stroke induces subregion-specific disruptions in thalamocortical FC, with distinct hyper-and hypoconnectivity profiles associated with motor and cognitive deficits. By mapping these network alterations to discrete thalamic nuclei, we provide a neuroanatomical framework for developing targeted neuromodulation therapies. Our findings underscore the pivotal role of the thalamus in post-stroke network reorganization, serving as a bridge between focal lesions and system-level dysfunction. However, this study has several limitations, including the modest sample size and lack of longitudinal follow-up data, which should be addressed in future investigations.

## Data Availability

The raw data supporting the conclusions of this article will be made available by the authors, without undue reservation.

## Footnotes

^1^ www.nitrc.org/projects/mricron
